# Identification of Gait Abnormalities in Patients with Hip Osteoarthritis Using a Monocular Vision 3D Motion Capture System

**DOI:** 10.3390/bioengineering13091066

**Published:** 2026-09-14

**Authors:** Kento Sabashi, Ryo Ueno, Mina Samukawa, Harukazu Tohyama

**Affiliations:** 1 Department of Rehabilitation, Hokkaido University Hospital, Kita 14, Nishi 5, Kita-Ku, Sapporo 060-8648, Hokkaido, Japan; 2 Department of Research and Development, ORGO Inc., 2-7, Odori Nishi 18, Chuo-Ku, Sapporo 060-0042, Hokkaido, Japan; 3 Department of Rehabilitation Science, Faculty of Health Sciences, Hokkaido University, Kita 12, Nishi 5, Kita-Ku, Sapporo 060-0812, Hokkaido, Japan; 4 Department of Orthopaedic Sports Science, Faculty of Health Sciences, Hokkaido University, Kita 12, Nishi 5, Kita-Ku, Sapporo 060-0812, Hokkaido, Japan

**Keywords:** hip osteoarthritis, gait abnormality, kinematic, artificial intelligence, video, motion capture

## Abstract

This study aimed to compare the accuracy of video-based kinematic measurements using artificial intelligence (AI) between patients with hip osteoarthritis (OA) and healthy participants, and to compare the gait abnormality estimated from video data with that calculated using optical three-dimensional motion capture (MOCAP) data. This study used open-access datasets of MOCAP and video data including 20 patients with hip OA and 20 healthy participants. Video data were processed using the MYoACT application to extract marker data. The mean absolute error (MAE) of joint angles between MOCAP and MYoACT data was calculated. In patients with hip OA, the modified Gait Abnormality Score (mGAS) for each joint angle was calculated by averaging the absolute differences between each patient’s value and the mean value of healthy participants, divided by the standard deviation of healthy participants, across the entire gait cycle. No significant difference in the MAE was observed between groups. In patients with hip OA, the mGAS showed no significant difference between MYoACT and MOCAP data. These findings suggest that AI-driven video-based kinematic measurements show no clear difference in the accuracy between patients with hip OA and healthy participants, and demonstrate the potential to identify gait abnormalities.

## 1. Introduction

Hip osteoarthritis (OA) causes structural changes in the hip joint [1] and leads to hip pain [2], reduced range of motion [3], and muscle weakness [4]. As a result, hip OA reduces physical function and quality of life (QOL) [5,6]. Because the number of patients with hip OA is expected to increase in the future [7], hip OA is one of the conditions that require further advancements in treatment strategies.

While previous research has identified several factors influencing physical function and QOL in patients with hip OA, such as hip muscle cross-sectional area [8], hip flexion range of motion [9], and walking speed [10], accumulating evidence suggests that gait abnormalities are associated with physical function and QOL in patients with hip OA. Riglet et al. [11] reported that a reduced hip extension angle during gait in patients with hip OA was associated with worsening physical function and QOL after three years. Additionally, Rosenlund et al. [12] quantified gait abnormalities in patients with hip OA using the Gait Deviation Index (GDI) and demonstrated a significant correlation between the GDI and both physical function and QOL in patients with hip OA. Therefore, quantitative gait analysis of patients with hip OA is crucial for rehabilitation. However, an optical three-dimensional motion capture (MOCAP) has not been easy to implement in clinical practice because it is costly and requires substantial time for data collection and analysis [13].

Recent advances in artificial intelligence (AI) have enabled simple and accurate pose estimations from smartphone videos, leading to high expectations for the clinical application of these technologies. To date, many studies have focused on validating the accuracy of these methods in healthy participants. Viswakumar et al. [14] calculated joint angles using the OpenPose from video data captured with a single smartphone and evaluated the accuracy by calculating the mean absolute error (MAE) relative to the MOCAP. As a result, the MAE values between the OpenPose and MOCAP were reported as 7.13° for ankle dorsiflexion, 5.82° for knee flexion, and 7.73° for hip flexion. Furthermore, Uhlrich et al. [15] demonstrated that the average MAE value between the OpenCap, which estimates joint angles from videos captured by two or more smartphones, and MOCAP was 4.5° across all joint angles. However, as these studies were conducted on healthy participants, it remains unclear how accurately kinematic measurements can be obtained in patients with hip OA who exhibit gait abnormalities [16,17]. Furthermore, it is unknown whether the evaluation of gait abnormalities, such as the GDI, yields significantly different results between the video-based pose estimation and MOCAP. Although the GDI is a composite measure that integrates all joint angles [18], evaluation based on individual joint angles is more practical for guiding interventions in clinical practice. Recently, a standardized method called the modified Gait Abnormality Score (mGAS), which evaluates gait abnormalities based on individual joint angles, has been developed [19]. In clinical practice, the mGAS is more practical than the GDI for gait assessment. Therefore, the aims of this study were twofold: first, to investigate whether the error in AI-driven video-based joint angles relative to the MOCAP differs between patients with hip OA and healthy participants; and second, to investigate whether the mGAS estimated from video data yields significantly different results to that calculated using the MOCAP.

## 2. Materials and Methods

### 2.1. Datasets

This study used open-access motion-capture datasets provided by Bertaux et al. [20] MOCAP and video data from 20 patients with unilateral hip OA (from 30 days to 1 day before total hip arthroplasty) and 20 healthy participants were used as experimental data (Table 1). Bertaux et al. recorded gait data using eight optoelectronic cameras (Vicon MXT40, Vicon Motion Systems Ltd., Oxford, UK) sampled at 100 Hz and a video camera (Basler camera) sampled at 50 Hz in the frontal plane [20]. A total of 35 reflective markers were attached to the whole body based on the Plug-in-Gait model [21]. The participants performed overground walking along a 6-m walkway as naturally as possible while looking forward.

### 2.2. Data Processing

**Figure 1:**
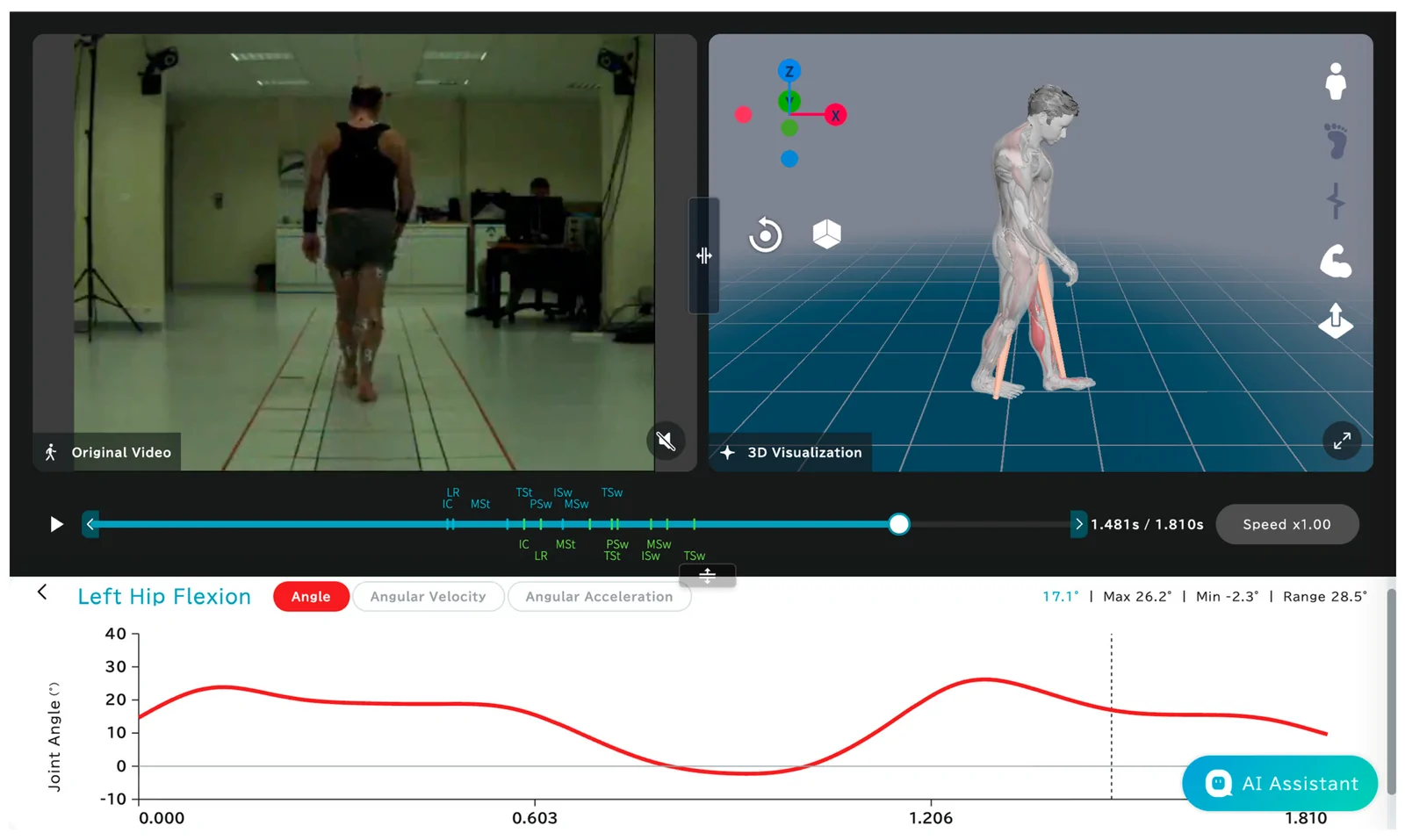
MYoACT is an application that enables AI-driven video-based musculoskeletal analysis including joint angles, ground reaction force, and joint moments.

MOCAP data were obtained from the recorded C3D files. Frontal plane video data were extracted as C3D files using the MYoACT 1.0.0 (ORGO Inc., Sapporo, Japan) (Figure 1). The MYoACT is an application that enables musculoskeletal analysis from video data using AI, including the estimation of joint angles, ground reaction forces, and joint moments. In principle, a previously described methodology is applied in the MYoACT [22,23]. Subsequently, the C3D files of MOCAP and MYoACT data were processed using CusToM, a MATLAB toolbox, to calculate the hip flexion, hip adduction, hip internal rotation, knee flexion, and ankle dorsiflexion angles [22,23,24]. Additionally, trunk and pelvic obliquity angles were calculated from the marker data. The trunk obliquity angle was defined as the frontal plane projection angle between the vertical axis and the line connecting the midpoints of the bilateral acromioclavicular joints and the midpoints of the bilateral anterior superior iliac spines [25]. The pelvic obliquity angle was defined as the frontal plane projection angle between the horizontal axis and the line connecting the bilateral anterior superior iliac spines [25]. Positive trunk and pelvic obliquity angles indicate an inclination toward the stance limb. Joint angles were analyzed for the affected side in patients with hip OA and a randomly selected side in healthy participants. The following 20 markers were used to calculate joint angles for both MOCAP and MYoACT data: the second metatarsal head, calcaneus, lateral malleolus, flexion–extension axis of the knee, posterior superior iliac spine, anterior superior iliac spine, acromioclavicular joint, spinous processes of the 7th cervical and 10th thoracic vertebrae, back of the head, and temple. All markers were attached bilaterally, except for the spinous processes of the 7th cervical and 10th thoracic vertebrae. Initial contact during gait was identified based on the contralateral peak hip extension angle, which was used to determine the gait cycle for both patients with hip OA and healthy participants [26].

To evaluate the accuracy of joint angles obtained from the MYoACT, the mean signed error (MSE) and mean absolute error (MAE) of MYoACT data relative to MOCAP data over one gait cycle was calculated for both patients with hip OA and healthy participants [27]. The MSE and MAE were calculated as follows:

$$MSE=\frac{1}{N}\sum_{i=1}^{N}\left(MOCAP_{i}-MYoACT_{i}\right)$$

$$MAE=\frac{1}{N}\sum_{i=1}^{N}\left|MOCAP_{i}-MYoACT_{i}\right|$$

where N = total number of time points, i = each time point, MOCAP = MOCAP data, and MYoACT = MYoACT data.

To evaluate gait abnormalities in patients with hip OA, the mGAS was computed separately for MOCAP and MYoACT data based on the concept of z-scores [28]. The mGAS indicates the extent to which the data of patients with hip OA deviates from the mean value of healthy participants, expressed in units of the standard deviation (SD). The mGAS was calculated by averaging the absolute differences between each patient’s kinematic value and the mean kinematic value of healthy participants, divided by the SD of kinematic values in healthy participants, across the entire gait cycle [19]. The kinematic values of healthy participants used for the calculation of the mGAS were obtained separately from the MOCAP and MYoACT data. This formula provides a quantitative evaluation of deviations from normal gait patterns. The formula is as follows:

$$mGAS=\frac{1}{N}\sum_{i=1}^{N}\left|\frac{P_{i}-\bar{H_{i}}}{\sigma_{i}}\right|$$

where P = each patient’s kinematic value, $\bar{H}$ = mean kinematic value of healthy participants, σ = SD of kinematic values in healthy participants.

### 2.3. Statistical Analysis

An unpaired *t*-test was performed to compare age, height, weight, and MAE between patients with hip OA and healthy participants. Regarding the MAE, an analysis of covariance (ANCOVA) was also performed, adjusting for factors that showed significant differences between groups in demographic data. A paired *t*-test was performed to compare the mGAS between MOCAP and MYoACT data in patients with hip OA. A chi-square test was used to compare the sex distribution between patients with hip OA and healthy participants. Statistical significance was defined as a *p* value < 0.05.

## 3. Results

Patients with hip OA were significantly older and heavier than healthy participants, whereas no significant differences were observed in height or sex (Table 1).

**Figure 2:**
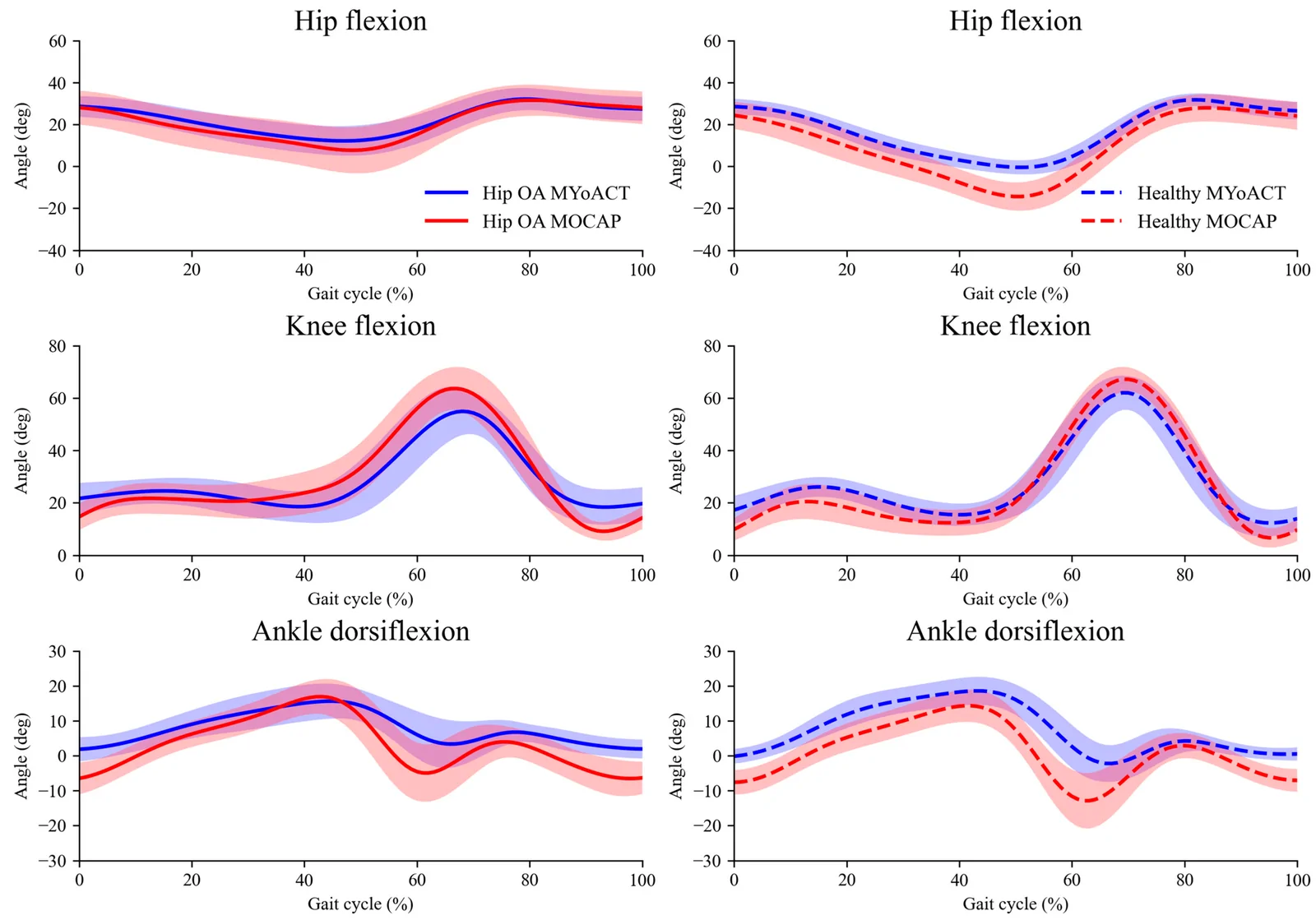
Mean kinematic waveforms of MYoACT and MOCAP data in patients with hip OA and healthy participants.

The mean kinematic waveforms obtained from MYoACT and MOCAP data for patients with hip OA and healthy participants are shown in Figure 2. The MSE values for patients with hip OA and healthy participants are presented in Table 2. The MAE values between MYoACT and MOCAP data showed no significant differences between patients with hip OA and healthy participants in any of the evaluated kinematic variables (Table 3). In patients with hip OA, no significant differences were observed in the mGAS between MYoACT and MOCAP data for any of the evaluated kinematic variables (Table 4).

## 4. Discussion

This study compared the accuracy of AI-driven video data relative to MOCAP data between patients with hip OA and healthy participants, and the mGAS in patients with hip OA between video and MOCAP data. The MAE value of all video-based joint angles relative to MOCAP data was 5.3° in patients with hip OA and 5.5° in healthy participants, with no significant difference between the two groups. Furthermore, no significant differences were found between the mGAS calculated from video data and those calculated from MOCAP data across all joints in patients with hip OA. These results indicate that video-based kinematic measurements using AI may identify the characteristics of patients with hip OA. AI-driven video-based kinematic measurements have the potential to become a valuable tool in clinical practice for patients with hip OA.

This is the first study to investigate the accuracy of joint angles and the mGAS during gait in patients with hip OA using video data recorded from a single camera. Viswakumar et al. [14] reported that joint angle errors between the OpenPose using a single camera and MOCAP in healthy participants were 7.13° for ankle dorsiflexion, 5.82° for knee flexion, and 7.73° for hip flexion. Additionally, Uhlrich et al. [15] reported that joint angle errors between the OpenCap using two or more smartphones and MOCAP in healthy participants was 4.5° across all joint angles. There was no significant difference in our results between patients with hip OA and healthy participants. Our findings suggest that the accuracy of video-based kinematic measurements using AI may be maintained even in patients with hip OA. An MAE value of 5° has been suggested as clinically acceptable [29,30]. Although several joint angles in the present study exhibited MAE values exceeding 5°, the mean MAE value across all joint angles was approximately 5° in patients with hip OA. Additionally, no significant difference was observed between AI-driven video and MOCAP data in the mGAS evaluating gait abnormalities. AI-driven video-based kinematic measurements may be capable of identifying gait abnormalities in patients with hip OA.

Exercise therapy is a common conservative treatment for patients with hip OA. Osteoarthritis Research Society International (OARSI) strongly recommends strengthening, cardio, and/or balance training as a core treatment [31]. Similarly, the European Alliance of Associations for Rheumatology (EULAR) recommendations support strength, aerobic, flexibility, or neuromotor exercises [32]. However, gait training (e.g., gait modification) has not been addressed in these guidelines. Riglet et al. [11] and Rosenlund et al. [12] demonstrated that gait patterns in patients with hip OA was associated with both physical function and QOL. Therefore, incorporating gait training in addition to conventionally recommended exercise therapies may further improve clinical outcomes. Although quantitative gait evaluation has conventionally required the MOCAP, its clinical implementation has remained limited due to its high cost and the long time required for data collection and analysis [13]. This study demonstrates the favorable accuracy of AI-driven video-based kinematic measurements in patients with hip OA and suggests its potential utility in extracting gait abnormalities. Consequently, AI-driven video-based kinematic measurements for hip OA patients holds great promise for future clinical practice.

There are several limitations to this study. First, because the participants included only 20 patients with hip OA and 20 healthy participants, the results may not be generalizable. Therefore, future research involving a larger sample size is required. Second, the kinematic values of the healthy participants used to calculate the mGAS were separately defined for MYoACT and MOCAP data. Therefore, the mGAS calculated from MYoACT data does not directly indicate deviations from normal gait patterns based on MOCAP data. Third, as this study estimated joint angles using only frontal plane video data from the database [20], it remains unclear whether the camera position was optimal. Finally, it should be noted that the hip extension angle was used to identify the initial contact during gait. Although the accuracy of this method for identifying initial contact has been reported in healthy participants [26], its accuracy in patients with hip OA remains unclear.

## 5. Conclusions

The accuracy of AI-driven video-based joint angles relative to the MOCAP did not significantly differ between patients with hip OA and healthy participants. Furthermore, regarding the evaluation of gait abnormalities in patients with hip OA, the mGAS calculated from video data using AI showed no significant difference from that calculated from the MOCAP. These findings suggest that AI-driven video-based kinematic measurements in patients with hip OA have the potential to be useful in rehabilitation.

## Figures and Tables

**Table 1:** Demographic data of patients with hip OA and healthy participants.

	Hip OA	Healthy	*p* Value
Age, years	62.0 (9.4)	51.6 (11.4)	0.003 *
Height, cm	167.5 (10.5)	167.3 (9.9)	0.939
Weight, kg	83.7 (15.5)	71.9 (15.2)	0.019 *
Sex (male/female), *n*	12/8	8/12	0.206
Kellgren–Lawrence grade, *n*			
Grade 2	4		
Grade 3	9		
Grade 4	7		

Data are presented as mean (SD). * *p* value < 0.05.

**Table 2:** MSE values of joint angles between MYoACT and MOCAP data in patients with hip OA and healthy participants.

	Hip OA	Healthy
Trunk obliquity, °	1.7 (1.6)	0.8 (2.1)
Pelvic obliquity, °	−1.1 (2.4)	0.6 (2.1)
Hip flexion, °	1.9 (8.4)	7.0 (6.9)
Hip adduction, °	2.4 (1.8)	2.9 (1.7)
Hip internal rotation, °	3.2 (4.8)	6.8 (3.9)
Knee flexion, °	−1.2 (5.3)	1.4 (5.3)
Ankle dorsiflexion, °	4.5 (5.5)	6.4 (5.2)

Data are presented as mean (SD).

**Table 3:** MAE values of joint angles between MYoACT and MOCAP data in patients with hip OA and healthy participants.

	Hip OA	Healthy	*p* Value	Adjusted *p* Value
Trunk obliquity, °	2.1 (1.1)	1.8 (1.3)	0.480	0.659
Pelvic obliquity, °	2.6 (1.1)	2.4 (0.8)	0.513	0.972
Hip flexion, °	7.5 (4.3)	8.9 (4.7)	0.328	0.315
Hip adduction, °	3.5 (1.2)	4.2 (1.2)	0.100	0.072
Hip internal rotation, °	5.7 (2.9)	7.5 (3.1)	0.067	0.052
Knee flexion, °	8.5 (3.5)	6.8 (2.6)	0.096	0.206
Ankle dorsiflexion, °	6.9 (3.0)	7.3 (4.4)	0.764	0.518
All joint angles, °	5.3 (1.0)	5.5 (1.9)	0.533	0.570

Data are presented as mean (SD). *p* value was calculated using an unpaired *t*-test. Adjusted *p* value was calculated using an ANCOVA, adjusting for age and weight.

**Table 4:** mGAS derived from MYoACT and MOCAP data in patients with hip OA.

	MYoACT	MOCAP	*p* Value
Trunk obliquity	1.22 (0.49)	1.02 (0.46)	0.090
Pelvic obliquity	0.97 (0.46)	1.32 (0.83)	0.106
Hip flexion	1.94 (0.95)	1.83 (0.84)	0.646
Hip adduction	1.34 (0.83)	1.15 (0.96)	0.241
Hip internal rotation	0.89 (0.57)	0.95 (0.72)	0.745
Knee flexion	1.10 (0.47)	1.42 (0.64)	0.087
Ankle dorsiflexion	1.14 (0.40)	1.03 (0.50)	0.434

Data are presented as mean (SD).

## Data Availability

The data presented in this study are available in Figshare at https://doi.org/10.6084/m9.figshare.14420645 and https://doi.org/10.6084/m9.figshare.14420756 [20].

## Abbreviations

The following abbreviations are used in this manuscript:

AI	Artificial intelligence
OA	Osteoarthritis
MOCAP	Motion capture
MAE	Mean absolute error
mGAS	Modified Gait Abnormality Score
QOL	Quality of life
GDI	Gait Deviation Index
MSE	Mean signed error
SD	Standard deviation
ANCOVA	Analysis of covariance
OARSI	Osteoarthritis Research Society International
EULAR	European Alliance of Associations for Rheumatology
